# Significance of Extranodal Extension in Surgically Treated HPV-Positive Oropharyngeal Carcinomas

**DOI:** 10.3389/fonc.2020.01394

**Published:** 2020-08-11

**Authors:** Anna Beltz, Stefanie Zimmer, Ioannis Michaelides, Katja Evert, Georgios Psychogios, Christopher Bohr, Julian Künzel

**Affiliations:** ^1^Department of Otorhinolaryngology, University Medical Center Mainz, Mainz, Germany; ^2^Institute of Pathology, Tissue Bank, University Medical Center Mainz, Mainz, Germany; ^3^Department of Otorhinolaryngology, University Hospital Regensburg, Regensburg, Germany; ^4^Institute of Pathology, University of Regensburg, Regensburg, Germany; ^5^Department of Otorhinolaryngology, University Hospital of Ioannina, Ioannina, Greece

**Keywords:** extranodal extension, TNM classification, human papilloma virus, oropharyngeal carcinoma, HPV, OPSCC

## Abstract

Squamous cell carcinomas of the head and neck are the subject of numerous current studies, especially in view of the increasing incidence of tumors induced by human papillomavirus (HPV) and the latest changes to the TNM classification of oropharyngeal squamous cell carcinoma (OPSCC). In addition to HPV status, the presence of extranodal extension of lymph node metastases represents an important risk and prognostic factor, which has now been integrated into the staging algorithm of the eighth edition of TNM classification for HPV-negative OPSCC. In the past numerous studies had shown a lack of prognostic significance of extranodal extension in HPV-associated tumors. However, extranodal extension–as a possible risk factor even in HPV-positive OPSCC–remains an important subject of current studies, which are now particularly characterized by high numbers of cases. In this paper, diagnostic methods and the prognostic significance of extranodal extension in surgically treated HPV-positive OPSCC are presented and discussed based on relevant literature, and the results of current publications are summarized. Further development of diagnostic criteria and procedures as well as international standardization of clinical diagnostics of extranodal extension should be encouraged. Several studies demonstrate that extranodal extension results in worse survival outcomes even in HPV-positive tumors, in contrast to results of previous studies. Consequently, whether the prognostic significance of extranodal extension is not actually relevant to outcome and the staging algorithm of HPV-positive OPSCC should be questioned and further analyzed.

## Introduction

The role of HPV in OPSCC has gained a great deal of attention in recent years. In addition to its causative role, HPV infection also proved to have a clear prognostic value (1). With the introduction of the eighth edition of the TNM classification (2017) a distinction is being made for the first time between HPV-positive and HPV-negative squamous cell carcinomas by the use of p16 immunohistochemistry (p16 IHC) as part of the staging of oropharyngeal squamous cell carcinoma (OPSCC). Furthermore, the prognostic influence of extranodal extension (ENE) of lymph node metastases for HPV-negative OPSCC was integrated into the staging algorithm. The prognostic influence of ENE has been analyzed in several studies and it was recognized as an essential prognostic factor, which should facilitate an even more accurate estimate of the risk of regional disease recurrences or distant metastases (1, 2). In the clinical staging of lymph node metastasis, defined criteria must be fulfilled for the diagnosis of clinical ENE. According to the new edition of the TNM classification, ENE in p16-positive OPSCC compared to HPV-negative tumors does not result in prognostic upstaging with regard to the N category or UICC stage. Several current studies focus on evaluating extensively the prognostic influence of ENE in HPV-positive tumors. The diagnostic methods and significance of ENE—with particular attention to surgically treated HPV-positive OPSCC—are presented and discussed below.

## Definition of Extranodal Extension

**Figure 1:**
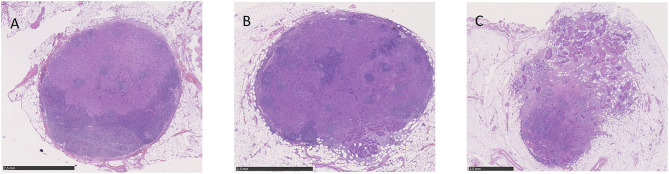
Microscopic view of lymph node metastases without ENE **(A)**, with microscopic ENE **(B)** and with macroscopic ENE **(C)**.

Extranodal extension was first described in 1930 by Rupert A. Willis in the context of autopsies on patients with advanced head and neck squamous cell carcinoma (HNSCC) (1, 3). It is generally defined as the spread of tumor tissue or neoplastic cells outside the lymph node capsule with infiltration of perinodal soft tissue (4). By means of histopathologic examination (lymph node metastasis without ENE; Figure 1A), ENE can additionally be subdivided into the categories “microscopic” (≤2 mm beyond the lymph node capsule; Figure 1B) and “macroscopic” (>2 mm beyond the lymph node capsule; Figure 1C) (4). For instance, Bauer et al. in their 2019 study illustrated the importance of the extent of ENE in patients with OPSCC (5). Patients were classified into the categories ENE-negative, microscopic ENE, and macroscopic ENE, and patients with microscopic ENE showed significantly reduced survival compared to patients with negative ENE status (hazard ratio = HR = 1.52; 95% CI = 1.00–2.31; *p* = 0.048) (5). Patients with macroscopic ENE had the worst outcome (HR = 2.50; 95% CI = 1.39–4.51; *p* = 0.002) (5). In addition to that, recent data even differentiate between the categories “no ENE,” “minimal ENE” (≤1 mm beyond the lymph node capsule”), and “>1 mm beyond the lymph node capsule” (6).

Although these subcategories have not yet been applied for the purpose of pN classification, they are recommended by the AJCC for data collection and analyses and find application in recent studies (4). In addition to patients with diagnosed lymph node metastases and ENE of metastases, it is reported that a proportion of 10.5–25% of patients exhibit microscopic ENE despite having a clinically unremarkable lymph node status (1, 7). Thus, microscopic ENE, micrometastases, or soft tissue deposits can cause an underestimate of the incidence of ENE—especially with regard to patients with primary radiotherapy that are classified within the cTNM-classification system (1, 7).

## Imaging and Clinical Predictors in Diagnosing Extranodal Extension

In addition to the widely used diagnostic methods involved in postoperative histopathologic examination, various imaging techniques can be applied, such as ultrasound, magnetic resonance imaging (MRI), and computed tomography (CT). Clinical diagnosis of ENE of lymph node metastases presupposes that clear, defined criteria are met (8). Clinical or radiologic signs of tumor invasion alone (including the skin and surrounding soft tissue) as well as clinical symptoms of neural involvement (e.g., paresis of cranial nerves) are defined as clinical ENE in the new TNM classification (9). The following criteria are used for radiologic diagnosis of ENE for both CT and MRI: presence of irregular nodal capsular enhancement, loss of distinct nodal margins and infiltration into adjacent structures (fatty tissue, muscle, blood vessels) (1, 10, 11). Generally speaking, however, the limited sensitivity and specificity of the methods used in relation to the clinical diagnosis of ENE need to be discussed (8). For example, Steinkamp et al. showed in several publications a sensitivity of ~80.9% with specificity of 72.2% for CT investigations and sensitivity of ~74.4% and specificity of 72.2% for MRI imaging (1, 12, 13). Clinical diagnosis of ENE by ultrasound, with a sensitivity of 78.6% and specificity of 81.8%, achieved slightly better results than CT or MRI (1, 14). With regard to diagnosing ENE by contrast-enhanced CT imaging, the values quoted in the literature according to Faraji et al. and others range from 75 to 86% for the accuracy of predicting pathologic ENE, from 65 to 90% for sensitivity and 73–91% for the specificity of the imaging method (12, 15–21). As clinical diagnosis of ENE, for example, does not differentiate between microscopic and macroscopic ENE so far, the data of patient collectives with primary surgery and collectives with primary radiotherapy are not readily comparable.

For a long time there have been strong demands for standardization and further development of investigation methods and internationally recognized diagnostic criteria for the imaging modalities of ultrasound, CT and MRI (1). Only recently Kann et al. published their study on the diagnosis of lymph node metastases and ENE in HNSCC by means of pretreatment CT images and three-dimensional deep learning neural networks (22). In this study they trained the neural network using a data set of 2,875 CT-segmented lymph node specimens and achieved diagnostic results which exceeded those of human clinicians (22). The area under the receiver operating characteristics curve for diagnosing ENE and lymph node metastases was 0.91 (95% CI = 0.85–0.97)—ENE of lymph node metastases could be predicted with a sensitivity of 88% and specificity of 85% (22). The diagnosis of ENE by means of CT was additionally the subject of the recently published work by Faraji et al. (15). Seventy-three patients with HPV-positive OPSCC treated by primary surgery and neck dissection were reviewed for the presence of seven defined criteria of CT imaging (15). The pretreatment CT scans were evaluated by two radiologists who were blinded to the pathologic ENE results (15). In the evaluations, the presence of irregular nodal margins (highest specificity of 94% for examiner A and 95% for examiner B) and the absence of perinodal fatty tissue (highest sensitivity of 87% for examiner A and 96% for examiner B) showed a significant association with ENE (15).

In 2018 Hararah et al. published initial attempts at pretreatment prediction of ENE and positive surgical margins for OPSCC (23). In the course of analyzing prognostic parameters of 5,056 patients (3,336 HPV-positive), Hararah et al. developed nomograms for the parameters ENE and/or positive resection status for HPV-negative as well as HPV-positive OPSCC (23). Regarding the prediction of postoperative ENE, for HPV-positive tumors clinical ENE, cN staging, cT staging, age, and tumor grading were integrated into the nomogram as predictive parameters (AUC ROC = 0.66; *p* < 0.01; 95% CI = 0.64–0.68) (23). Hararah et al. are thus presenting additional approaches to diagnosing ENE which, as a whole, could potentially facilitate clinical decision-making regarding primary treatment. However, they particularly stress the current aspiration to further develop clinical (and pathologic) diagnostic methods.

Further studies and more prolonged use of the new TNM classification are required to show how far the new clinical N classification, or particularly the clinical diagnosis of ENE defined therein, can succeed in everyday clinical practice and result in reliable identification of ENE status or whether new diagnostic methods will prevail in future. Understaging or upstaging of patients not surgically treated should be avoided where the prognostic influence of ENE is proven. Improving the modalities for clinical diagnosis of ENE and standardizing ENE diagnosis in general will thus continue to be the objective over the next few years.

## Methods

**Figure 2:**
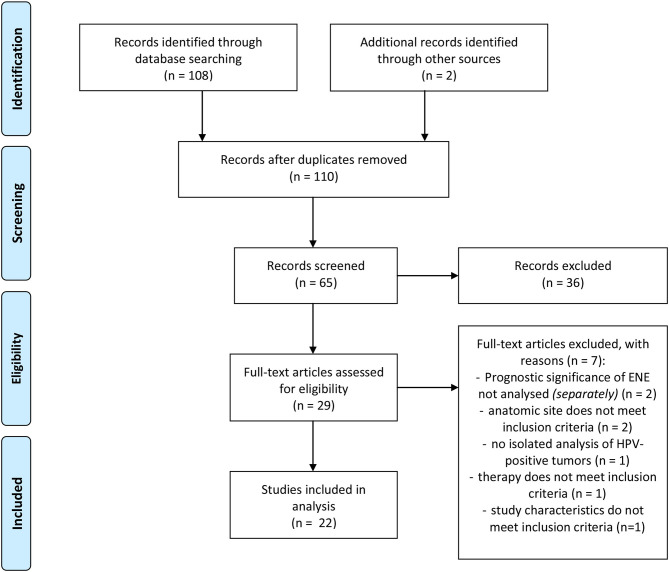
Flow-chart of literature research adapted from PRISMA (24).

The aim of this paper is to provide a structured overview of current study results on the topic “Prognostic influence of ENE in surgically treated HPV-positive OPSCC.” In order to investigate a possible prognostic influence of ENE in HPV-positive OPSCC, a literature research was conducted with *PubMed*. Using the *PubMed Search Builder* the following search term was created: ((((((((((extracapsular spread[Title/Abstract]) OR perinodal spread[Title/Abstract]) OR transcapsular spread[Title/Abstract]) OR extranodal spread[Title/Abstract]) OR extracapsular extension[Title/Abstract]) OR extranodal extension[Title/Abstract]) OR perinodal extension[Title/Abstract]) OR transcapsular extension[Title/Abstract])) AND (((hpv[Title/Abstract]) OR human papilloma virus[Title/Abstract]) OR p16[Title/Abstract])) AND (((((head and neck squamous cell carcinoma)) OR hnscc) OR oropharyngeal squamous cell carcinoma) OR opscc). The process of literature research is illustrated in Figure 2. 109 of 110 results were available in English. The included studies were published before July 2020.

Before screening the records, the following inclusion criteria were defined: (a) OPSCC, (b) patient collective contains HPV-positive tumors, (c) surgically treated collective/Neck Dissection (with or without adjuvant therapy), (d) ENE-Status available, (e) statement on the prognostic influence of ENE, (f) original research paper. Full-text articles assessed for eligibility were screened for further relevant publications. The publications included in analysis were screened for their study results referring to the prognostic impact of ENE in HPV-positive OPSCC. The relevant results as well as characteristics of the studies and number of cases are shown in Tables 1, 2 in the following.

**Table 1:** Summary of the described studies on the prognostic influence of ENE in HPV-positive OPSCC—part 1.

**References**	**Characteristics**	**Patients**	**Prognostic influence of ENE in HPV+ OPSCC**
Lewis et al. (25)	− Monocentric − Retrospective − Surgical	*n* = 101 OPSCC (HPV+: *n* = 90)	− Univariate analysis: only grade 4 ENE (soft tissue deposit) associated with OS (*p* < 0.001), DFS (*p* = 0.0025), and DSS (*p* = 0.0013) (but correlates with T stage) − Multivariate analysis: no significant correlation with OS, DFS, or DSS
Haughey et al. (26)	− Monocentric − Retrospective − Surgical	*n* = 171 OPSCC (HPV+)	ENE not significantly associated with OS, DSS, or DFS
Sinha et al. (27)	− Monocentric − Prospective − Surgical	*n* = 152 OPSCC (HPV+)	− Univariate analysis: grade 4 ENE (soft tissue deposit) significantly associated with DFS (*p* = 0.02), DSS (*p* = 0.03), and OS (*p* = 0.009) − Multivariate analysis: grade 4 ENE (soft tissue deposit) significantly associated with DFS (*p* = 0.01), DSS (*p* = 0.01), and OS (*p* = 0.03) − ENE grade 0–3 not prognostic
Klozar et al. (28)	− OPSCC+OSCC − Monocentric − Retrospective − Surgical (except for 3 patients)	*n* = 139 OPSCC (HPV+: *n* = 91) *n =* 31 OSCC (HPV*+: n =* 7)	ENE in univariate and multivariate analysis not significantly associated with DSS
Maxwell et al. (29)	− OPSCC+OSCC − Monocentric − Retrospective − Surgical	*n* = 133 OPSCC (HPV+: *n* = 76) *n =* 214 OSCC	− OPSCC: ENE not significantly associated with DSS (*p* = 0.936) (also for HPV-OPSCC, *p* = 0.198) − ENE as significant independent prognostic factor in OSCC
Sinha et al. (30)	− Monocentric − Prospective − Surgical	*n* = 220 OPSCC (HPV+)	− ENE not prognostic for DFS, DSS, or recurrence − Number of lymph node metastases significantly associated with outcome
Sinha et al. (31)	− Monocentric − Prospective − Surgical	*n* = 222 OPSCC (HPV+)	− Grade 4 ENE (soft tissue deposit) significantly associated with distant metastases and DMFS (*p* = 0.004) only for T3–T4 tumors − No significant correlation with regional recurrence
Iyer et al. (32)	− Monocentric − Retrospective − Surgical	*n* = 201 OPSCC (HPV+: *n* = 106)	ENE not significantly associated with 5Y-OS (*p* = 0.300), 5Y-DSS (*p* = 0.116), or 5Y-RFS (*p* = 0.753)
Kaczmaret al. (33)	− (Monocentric) − Retrospective − Surgical	*n* = 114 OPSCC (HPV+)	Univariate analysis: ENE not significantly associated with increased risk of local and distant progression (*p* = 0.575, *p* = 0.793)
Kumar et al. (34)	− Monocentric − Retrospective − Surgical	*n* = 289 OPSCC (HPV+: n=172)	− Univariate analysis: ENE nearly reached significance (*p* = 0.0553) − Multivariate analysis: ENE not significantly associated with OS (*p* = 0.7644)
Kharytaniuk et al. (35)	− OPSCC+CUP − Monocentric − Retrospective − Surgical (neck dissection)	*n* = 62 OPSCC (HPV+: *n* = 36)*n =* 21 CUP (HPV*+: n =* 9)	− ENE not significantly associated with RFS (*p* = 0.93) or DSS (*p* = 0.91)
Tassone et al. (36)	− Monocentric − Retrospective − Surgical	*n* = 85 OPSCC (HPV+)	− Logistic regression analysis of recurrence (as binary variable): ENE not significantly associated with recurrence (OR = 2.28, *p* = 0.383) − Univariate analysis of DFS: ENE no significant impact on DFS (*p* = 0.25)

*CUP, cancer of unknown primary; DFS, disease-free survival; DMFS, distant metastasis-free survival; DSS, disease-specific survival; ENE, extranodal extension; HPV, human papillomavirus; HR, hazard ratio; NCDB, National Cancer Database; OPSCC, oropharyngeal squamous cell carcinoma; OR, odds ratio; OS, overall survival; OSCC, oral squamous cell carcinoma; RFS, recurrence-free survival*.

**Table 2:** Summary of the described studies on the prognostic influence of ENE in HPV-positive OPSCC—part 2.

**References**	**Characteristics**	**Patients**	**Prognostic influence of ENE in HPV+ OPSCC**
An et al. (37)	− NCDB (multicenter design) − Retrospective − Surgical	*n* = 1,043 OPSCC (HPV+)	− Univariate analysis: significant correlation with 3Y-OS (*p* = 0.01) − Multivariate analysis: significant correlation with OS (HR = 1.89; 95% CI = 1.01–3.51; *p* = 0.046) − Only patients with 1 lymph node metastasis: significant correlation with 3Y-OS (*p* = 0.033)
Zhan et al. (38)	− NCDB (multicenter design) − Retrospective − Surgical (neck dissection)	*n* = 3,745 OPSCC (HPV+)	− Univariate analysis: significant correlation with 4Y-OS (*p* < 0.001) − Also after stratification according to N classification for pN1 tumors
Shevach et al. (39)	− Monocentric − Retrospective − Surgical (neck dissection)	*n* = 75 OPSCC (HPV+)	− Univariate analysis: significant correlation with 5Y-DC rate (*p* = 0.046) and 5Y-PFS (*p* = 0.021) − Multivariate analysis: independently prognostic of worse DC (HR = 8.26; 95% CI = 1.24–55.21; *p* = 0.029) and PFS (HR = 4.64; 95% CI = 1.18–18.29; *p* = 0.028) − No significant difference in 5Y-LRC or OS
Meyer et al. (40)	− Monocentric − Retrospective − Surgical	*n* = 88 OPSCC (HPV+: *n* = 39)	− Univariate analysis: significant correlation with OS (*p* = 0.012) und RFS (*p* = 0.012) − Multivariate analysis: ENE not included
Bauer et al. (5)	− NCDB (multicenter design) − Retrospective − Surgical	*n* = 4,153 OPSCC (HPV+)	− Univariate analysis: significant correlation with 5Y-OS (*p* = 0 < 0.001) − Stratified according to N stage (8^th^ edition): significant correlation with 5Y-OS (*p* = 0 < 0.001) − Multivariate analysis: significant prognostic parameter (HR = 1.90; 95% CI = 1.35–2.67; *p* = 0 < 0.001)
Miccio et al. (41)	− NCDB (multicenter design) − Retrospective − Surgical	*n* = 3,407 OPSCC (HPV+)	− Univariate analysis: significant correlation with OS (HR = 2.04; 95% CI = 1.59–2.63; *p* < 0.001) − Multivariate analysis: significant correlation with OS (HR = 1.66; 95% CI = 1.26–2.19; *p* < 0.001)
Beltz et al. (42)	− Monocentric − Retrospective − Surgical	*n* = 95 OPSCC (HPV+: *n* = 50)	− Univariate analysis: significant correlation with 5Y-OS, (*p* = 0.008)
Han et al. (43)	− NCDB (multicenter design) − Retrospective − Surgical (alone)	*n* = 736 OPSCC (HPV+)	− Univariate analysis: presence of microscopic ENE (*p* = 0.009) or macroscopic ENE (*p* = 0.007) associated with increased risk of death − Multivariate analysis: macroscopic ENE vs. non-ENE as independent risk factor for death (HR = 4.9; 95% CI = 1.4–18.1; *p* = 0.016)
Freitag et. al. (44)	− Monocentric − Retrospective − Surgical	*n* = 92 OPSCC (HPV+)	− p16+: significant correlation with OS (*p* = 0.007) and TSS (*p* = 0.047) − p16+/HPV16 DNA+: significant correlation with OS (*p* = 0.013) and TSS (*p* = 0.026) − Multivariate analysis: independent predictor for decreased OS (*p* = 0.033), TSS (*p* = 0.165), PFS (*p* = 0.42), and DFS (*p* = 0.04)
Gal et al. (45)	− NCDB (multicenter design) − Retrospective − Surgical	*n* = 16,845 OPSCC (HPV+: *n* = 8,780)	− Pathologic and clinical ENE associated with decreased survival − No significant difference between pathologic and clinical ENE

*CI, confidence interval; DC, distant control DFS, disease-free survival; ENE, extranodal extension; HPV, human papillomavirus; HR, hazard ratio; NCDB, National Cancer Database; OPSCC, oropharyngeal squamous cell carcinoma; OS, overall survival; PFS, progression-free survival; RFS, recurrence-free survival; TSS, tumor-specific survival*.

## Extranodal Extension as Risk And Prognostic Factor

Numerous studies in the past have shown that the presence of ENE additionally worsens the prognosis of patients with HNSCC (1, 8, 46–50). In a 2006 meta-analysis by Dunne et al. involving 1,620 patients with diagnosed HNSCC and lymph node metastasization, 5-year overall survival deteriorated to 30.7% in the presence of ENE compared to 58.1% in the absence of ENE (1, 50).

Furthermore, the correlation between ENE and locoregional recurrence and distant metastases has been studied in recent years. For example, Myers et al. showed in their publication of 2001 on 266 patients with squamous cell carcinoma of the tongue that ENE was the most significant prognostic parameter for the risk of regional recurrence and distant metastases in their population (1, 2). The meta-analysis by Mermod et al. from 2016 showed an odds ratio of 2.18 (95% CI = 1.23–3.87) for the correlation between ENE and distant metastases (1).

In 2014 Künzel et al. analyzed, among other things, the influence of ENE on the disease-specific survival of patients with OPSCC not stratified to HPV status. The study analyzed 384 patients first diagnosed between 1980 and 2010 (48). One of the findings was that ENE is associated with significantly worse disease-specific survival of 50% compared to 81% in the absence of ENE (*p* < 0.001) (48).

A few studies, however, demonstrated a lack of significant worsening of outcome due to ENE in HPV-positive OPSCC (1, 25, 27, 29, 30). In particular, the research group of Sinha et al. investigated the influence of ENE in HPV-induced OPSCC in detail (1, 25–27, 30, 31). In 2012, with regard to a group of 171 patients with p16-positive surgically treated OPSCC (adjuvant RT: *n* = 73, adjuvant CRT: *n* = 69), they published a lack of significant impact of ENE on overall and disease-specific survival (1, 26). In their multivariate analysis from 2012 (27), ENE (except for soft tissue deposits) did not prove prognostic in HPV-positive OPSCC in a prospective transoral laser surgery database (*n* = 152 patients—adjuvant RT: *n* = 66, adjuvant CRT: *n* = 67) (1, 27). In 2015 in their multivariate analysis of p16-positive OPSCC treated by surgery and neck dissection (*n* = 220 patients—RT: *n* = 97, CRT: *n* = 75), one of their findings was that the number of lymph node metastases (≥5)—but not ENE—was an independent prognostic factor (1, 30).

Their analyses of 2011, 2012, and 2015 additionally investigated the significance of the extent of ENE and its prognostic influence: Thus Lewis et al. also described a lack of significant influence of ENE on overall survival, disease-free survival (DFS), and disease-specific survival (DSS) in surgically treated HPV-positive OPSCC (*n* = 101 patients–postoperative radiation therapy: *n* = 100, postoperative chemotherapy: *n* = 44) (25). In fact, a significant correlation was found between the presence of soft tissue deposits (defined as grade 4 ENE) and overall survival, DSS and DFS. Given a correlation with the T stage, it was not confirmed in the multivariate analysis (25). In their 2012 publication, Sinha et al. then confirmed a significant influence of soft tissue deposits on overall survival, DFS and DSS (27). In 2015 they again published results which showed a significant correlation between soft tissue deposits and distant metastasis-free survival for T3–T4 tumors only (*n* = 222 patients, adjuvant RT: *n* = 97, adjuvant CRT: *n* = 78) (31).

In this connection Maxwell et al. reached the following conclusion in their 2013 publication (29): In their analysis of 133 patients with OPSCC and 214 patients with carcinoma of the oral cavity (surgically treated in the years 1983–2009), they found no significant association between ENE status and DSS for HPV-positive and HPV-negative patients (OPSCC: adjuvant radiation: *n* = 111, adjuvant chemotherapy: *n* = 40).

The investigation of potential prognostic parameters of HPV-positive and HPV-negative OPSCC and oral carcinomas of 170 patients (OPSCC: *n* = 139, 65.5% HPV-positive) was also the purpose of the study by Klozar et al. published in 2013 (28). For HPV-negative tumors, univariate analysis showed UICC stage, Pt, and pN classification, number of positive lymph nodes and ENE to be significant prognostic parameters. Except for ENE, these were confirmed in the multivariate analysis. For HPV-*positive* tumors, by contrast, none of these parameters showed a significant correlation with the DSS of patients (28).

In 2015 Iyer et al. published the following results: While ENE, resection status, lymph vessel invasion, and pN category were independent predictors of survival in the case of HPV-negative OPSCC, they were not prognostic for HPV-positive tumors [in respect of recurrence-free survival (RFS), DSS, and OS] (*n* = 201 patients, adjuvant RT: *n* = 138) (32). In addition to that, Kumar et al. (34) showed that ENE (*p* = 0.0021) and advanced T-classification represent significant predictors of survival in HPV-negative surgically treated OPSCC (most patients treated with adjuvant RT/RCT based on NCCN guidelines). While ENE nearly reached significance in the univariate analysis of HPV-positive OPSCC (*p* = 0.0553), multivariate analysis revealed that ENE was not significantly associated with survival (*p* = 0.7644) (34). Kharytaniuk et al. in their 2016 analysis of 83 patients (*n* = 62 with OPSCC, *n* = 21 with cancer of unknown primary = CUP) with neck dissection as part of primary definitive treatment—also confirmed that ENE is not a negative prognostic factor for HPV-positive OPSCC (in respect of RFS and DSS) (35) (surgery only: *n* = 8, RT: *n* = 50, CRT: *n* = 25). Kaczmar et al. showed that ENE does not correlate with increased risk of local as well as distant progression in HPV-positive OPSCC (114 surgical patients, 89 with adjuvant radiation, 54 with adjuvant chemotherapy) in univariate analysis (33). In addition to that, Tassone et al. confirmed that ENE is not significantly associated with recurrence and DFS in a retrospective analysis of 85 surgically treated HPV-positive OPSCC (adjuvant RT: *n* = 81, adjuvant systemic therapy: *n* = 52) (36). Table 1 summarizes the studies described in this chapter showing no or weak prognostic influence of ENE in primarily surgically treated HPV-positive OPSCC.

## Extranodal Extension in the Context of the 8th UICC Classification

**Figure 3:**
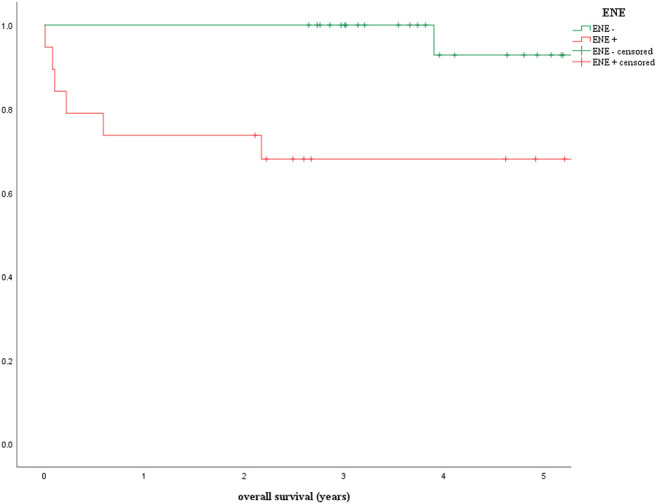
Prognostic influence of extranodal extension in patients with p16-positive oropharyngeal carcinoma (ENE, extranodal extension) (42).

According to the 8th edition of the TNM classification, the presence of ENE leads to distinct upstaging solely in HPV-negative OPSCC. For HPV-positive tumors, only the number of positive lymph nodes is decisive in terms of pTNM staging. This is why the prognostic influence of ENE in HPV-induced tumors is currently the focus of a number of studies. In a 2019 study by the present authors, the application and prognostic impact of the new TNM classification as well as the factors HPV status and ENE were examined in a group of 255 patients with OPSCC first diagnosed in the years 2008–2015 (42). This included analyzing the overall survival of HPV-positive patients with negative vs. positive ENE status treated with surgery alone or surgery combined with adjuvant radiation/chemoradiation. In this cohort adjuvant therapy was standard in case of pathologically proven ENE. This study addressed, among other things, the question of whether ENE can actually be ignored in HPV-mediated OPSCC (42). Ninety five patients met the inclusion criteria for ENE analysis. The Kaplan-Meier curves presented (Figure 3) and the log rank test revealed a statistically significant deterioration of overall survival in the presence of ENE for HPV-positive patients in the univariate analysis (ENE-negative: OS = 92.9%, ENE-positive: 68.0%, *p* = 0.008) (42).

The univariate analysis of the study by Meyer et al. (40), which examined the prognostic influence of the lymph node ratio, also showed a significant influence of ENE on overall survival of HPV-positive patients with surgically treated OPSCC (*p* = 0.012; ENE not included in the multivariate analysis) (surgery: *n* = 21, surgery + R(C)T: *n* = 67).

In 2017 Zhan et al. published results of their validation of the new staging system based on 3,745 cases of HPV-positive OPSCC treated by surgery and neck dissection from the National Cancer Database (NCDB) for the years 2010–2014 (surgery only: *n* = 642, surgery + RT: *n* = 1,005, surgery + CRT: *n* = 1,773) (38). As well as a general evaluation of the new staging algorithm, the study focused on analyzing the prognostic influence of ENE in HPV-positive OPSCC. During the course of their analyses, Zhan et al. demonstrated a statistically significant influence of ENE on 4-year overall survival in HPV-positive OPSCC (ENE-negative: 92% vs. ENE-positive: 85%, *p* < 0.001) (38). Upon stratification according to pN classification, ENE proved to be a significant prognostic parameter for the 4-year overall survival of HPV-positive patients with pN1 stage [pN1: ENE-negative 92%, ENE-positive 87% (*p* = 0.004); pN2: ENE-negative 88%, ENE-positive 77% (*p* = 0.061)] (38).

The described results are consistent with the results of analyses by An et al. published in 2017 (37): In their study the prognostic value of ENE was examined in a group of 1,043 patients with HPV-positive OPSCC (pT1–T4, pN1–N3, M0, R0) who underwent primary surgical treatment (adjuvant RT: *n* = 306, adjuvant CRT: *n* = 498). Patients who met the defined inclusion criteria were identified via the NCDB for the years 2010–2012 (37). In the course of their analyses An et al. demonstrated that a positive ENE status is associated with a significant deterioration of overall survival of HPV-positive patients (3-year overall survival: 89.3 vs. 93.6%, *p* = 0.01) (37). No significant difference in overall survival was found between cases with microscopic vs. macroscopic ENE (37). Furthermore, An et al. also demonstrated in the multivariate analysis that rather than the presence of ≥5 lymph node metastases—as integrated into the TNM classification for HPV-positive OPSCC—it is the presence of ENE that is significantly associated with a deterioration of overall survival (HR = 1.89; 95% CI = 1.01–3.51; *p* = 0.046) (37). Hence the results to some extent contradict the current system of N classification of p16-positive OPSCC (42). For HPV-positive OPSCC patients who have undergone primary surgical treatment, it is only the absolute number of lymph nodes involved (cut-off between pN1 and pN2: ≥5 involved lymph nodes) which has the decisive prognostic influence on determining the pN category according to the 8th edition (42). Given comparable hazard ratios (≥5 lymph node metastases: HR = 1.81, *p* = 0.086 vs. ENE: HR = 1.89, *p* = 0.046), An et al. do not question the cut-off of ≥5 lymph node metastases as a prognostic parameter for HPV-positive OPSCC. However, they do advocate evaluation of both parameters as potential prognostic factors—especially since a higher number of lymph node metastases is associated with the presence of ENE with a greater probability (37).

In order to eliminate the possible confounding variable of “total number of lymph node metastases” from the analysis and to investigate the relationship between ENE and overall survival in isolation, overall survival was analyzed only in patients with one lymph node metastasis: once again a deterioration of 3-year overall survival to 90.8% compared to 96.0% (*p* = 0.033) was observed (37).

In addition to that, Shevach et al. (39) published results showing that ENE-positive status is significantly associated with a deterioration of distant control and progression-free survival in univariate and multivariate analysis (39). They evaluated a collective of 75 patients with HPV-mediated OPSCC treated with surgery, respectively, neck dissection followed by adjuvant radiotherapy/chemoradiotherapy. However, there was no significant difference in OS and locoregional control between ENE-negative and -positive patients (39).

Bauer et al. recently showed that ENE represents a significant prognostic parameter in respect of overall survival in HPV-mediated OPSCC (5). In their paper published in 2019, they analyzed the prognostic significance of ENE in a group of 4,153 patients with HPV-positive OPSCC from the NCDB who were treated surgically and by neck dissection (N0 = 531, ENE-positive: 1,429, ENE-negative: 2,193—surgery only: *n* = 923, surgery/radiation: *n* = 1,403, surgery/radiation/chemo: *n* = 1,827) (5). The univariate analysis revealed a statistically significant correlation between ENE and overall survival in HPV-positive patients (*p* < 0.001) with 5-year overall survival of 92.6% (95% CI = 90.5–94.7%) for negative ENE status compared to 84.0% (95% CI = 80.7–87.4%) for ENE-positive tumors (5). Furthermore, when stratified according to N stage (8th edition), tumors classified as N1/ENE-negative showed the highest 5-year overall survival rate of 93.4% (95% CI = 91.3–95.5%), whereas N2/ENE-negative and N1/ENE-positive tumors had similar 5-year overall survival of 87.8 and 87.3%, respectively, (5). The multivariate analysis (with age, gender, population group, morbidity, T stage, treatment, and resection status as possible confounding variables) revealed in respect of mortality risk a hazard ratio of 1.90 (95% CI = 1.35–2.67) in the presence of ENE vs. ENE-negative OPSCC (*p* < 0.001). The pathologic N stage—or hence the number of positive lymph nodes—was significantly associated with patients' outcome (pN2 vs. pN1: HR = 1.53) (5). Furthermore, Bauer et al. demonstrated that—when combining pN category and ENE status—tumors classified as *N2/ENE-positive* had the lowest 5-year overall survival rate (HR = 2.93; 95% CI = 1.94–4.43; *p* < 0.001) in comparison with N1/ENE-negative OPSCC (HR = 1.00) (5). OPSCC classified as N1/ENE-positive also showed nearly twice as high mortality risk (HR = 1.88; 95% CI = 1.26–2.80; *p* = 0.002) as ENE-negative pN1 tumors. Bauer et al. thus concluded in the course of their evaluation that ENE is prognostic irrespective of the number of positive lymph nodes and that the combination of ENE status and number of positive lymph nodes (pN category) particularly leads to an improved picture of mortality risk (5). All in all, the work by Bauer et al. including 4,153 patients represents a large study in this field for HPV-positive OPSCC and—as a result of the length of follow-up—also allows the prognostic influence of ENE on 5-year overall survival to be evaluated. As Bauer et al. also stress, this is particularly significant in view of the relatively good prognosis of HPV-positive tumors. Furthermore, a large number of possible confounding variables were integrated into the multivariate analysis. In summary, the large number of cases included and the length of follow-up made it possible for the results to reach statistical significance (5).

Furthermore, Miccio et al. recently published their study on the influence of contralateral lymph node metastasization and ENE on survival in HPV-mediated OPSCC (41). Three thousand four hundred seven patients from the NCDB (2010–2015) with surgically treated, HPV-positive OPSCC and a minimum of 10 lymph nodes removed made up the study population (adjuvant RT: *n* = 1,262, adjuvant CRT: *n* = 1,501, unknown: *n* = 78) (41). In their evaluation, the research group of Miccio et al. concluded that, in both the univariate analysis (HR = 2.04; 95% CI = 1.59–2.63; *p* < 0.001) and the multivariate analysis (HR = 1.66; 95% CI = 1.26–02.19; *p* < 0.001), the presence of ENE is associated statistically highly significantly with a deterioration of overall survival in HPV-positive tumors and it should be included in future staging algorithms (41).

In 2019, Han et al. published a retrospective analysis of 736 patients with only surgically treated HPV-positive OPSCC from the NCDB (2010–2014) (43). Among other things, they showed that microscopic or macroscopic ENE results in a significantly worse OS when compared to positive lymph nodes without ENE (5J-OS: 91% vs. 78%; *p* < 0.0001) (43). In addition, Freitag et al. recently published their analysis of a cohort of 92 patients with surgically treated HPV-mediated OPSCC (IC+OP+RT: *n* = 8, OP: *n* = 21, OP+RT: *n* = 23, OP+RCT: *n* = 39, OP+RT+Cetuximab: *n* = 1) (44). Their multivariate analysis showed that ENE represents an independent predictor for decreased OS (*p* = 0.033), tumor-specific survival (*p* = 0.165), progression-free survival (*p* = 0.42), and DFS (*p* = 0.04) (44). The results of their investigation as a whole led them to the conclusion that ENE (as well as HPV16 DNA status) should be integrated in the prognostic staging algorithm of HPV-mediated OPSCC (44). Furthermore, Gal et al. recently showed a decreased survival in the presence of clinical and pathological ENE compared to the absence of ENE. Their retrospective SEER database study analyzed 16,845 primarily surgical treated patients with tonsillar and base of the tongue primaries (45).

As a whole the results described above contrast with the conclusions of the 2016 review by Mermod et al. which included an analysis of the prognostic significance of histopathologically proven ENE in HPV-positive OPSCC (1). Individual results of the studies included have already been presented: overall, the analyses had shown a lack of negative influence of ENE in HPV-positive OPSCC (1). Compared with the monocentric design of the studies analyzed by Mermod et al. (1) and the maximum number of 222 patients included Sinha et al. the great strengths of the studies by Zhan et al. (38), An et al. (37), Bauer et al. (5), Miccio et al. (41), and Gal et al. (45) are the case numbers of 3,745, 1,043, 4,153, 3,407, and 16,845 patients, respectively, and hence their power as well as their multicenter design.

Table 2 summarizes the described studies showing significant influence on prognosis of ENE in HPV-positive OPSCC.

The possible influence of tobacco consumption of patients was not analyzed because of the lack of recording in the NCDB. The influence of nicotine consumption on the risk and prognostic profile of HPV-positive OPSCC, however, is a relevant parameter according to the results of Ang et al. (51) and should be considered in future prospective analyses. Kompelli et al. recently published an analysis of patients with HPV-related OPSCC (52): Aim of this study was to investigate the impact of pathologic prognostic factors in the context of chronic tobacco use. The results show that, among other things, presence of ENE did not significantly affect survival in HPV-positive heavy smokers (≥20 pack years) (52). However, HPV-positive ENE-positive heavy smokers had a significant decrease in survival (similar to HPV-negative patients) compared to HPV-positive non-smokers with positive ENE-status (52). In the future, the prognostic impact of ENE should also be evaluated in the context of tobacco consumption and the prognostic influence of tobacco abuse in HPV-positive OPSCC should be examined in detail further on.

All in all, the current study results from various publications presented here emphasize that ENE is a risk and prognostic factor, including HPV-positive OPSCC, which to date has not been integrated into the staging algorithm of the TNM classification. Possible reasons for the different results of the studies mentioned are discussed by Bauer et al. (5), An et al. (37), and Zhan et al. (38). For example, the excellent prognosis of HPV-positive OPSCC could lead to the fact that only studies with a higher number of cases can reveal statistically significant differences between ENE-positive and—negative tumors (5). An et al. stress the greater power achieved by larger patient collectives as well (37). In addition, Zhan et al. support this argumentation with their data–with a moderate effect size on OS (5–11% on 4Y-OS), significant results are only likely in high numbers of cases (38), such as those made possible by the NCDB. Nonetheless, the prognostic influence of ENE in surgically treated HPV-positive OPSCC remains a topic that should be analyzed in further (prospective) multicenter studies.

Limitations of this work are that only studies that explicitly examined ENE in HPV-positive OPSCC (e.g., ENE terms in title/abstract) were included—therefore other possibly relevant research results, which were not identified through literature research or references, could have been missed. The aim of this publication was to provide the reader with an overview of the current status of research in this field in a structured form. Due to a limited number of studies that *explicitly* focus on this issue, as well as partly limited comparability, we did not choose a *systematic* review, but a structured review according to the PRISMA guidelines. We performed our literature research as structured and traceable as possible. Furthermore, no quality assessment of the included studies was carried out. Despite the focus on surgically treated collectives, it can be assumed that the therapy algorithms (especially regarding adjuvant therapy) vary to a certain extent from institute to institute, which influences the comparability of the studies. As histopathologically proven ENE is an accepted risk factor in HNSCC in general and de-escalation strategies in HPV-positive tumors have not been integrated into clinical practice, the majority of patients of all discussed studies should have been treated by adjuvant radiotherapy. As a topic that is currently gaining more and more interest, a future meta-analysis—including possible additional publications of the next months/years—should be considered.

## Conclusion

Whether the prognostic influence of ENE of lymph node metastases can actually be ignored for HPV-*positive* tumors in the TNM classification system should be reevaluated in detail in the context of prospective multicenter studies. According to the study results presented here, it also seems necessary to record ENE in the tumor documentation for HPV-positive tumors. Furthermore, it is also the extent of ENE (macroscopic vs. microscopic—ENE ≤/>1 mm, respectively, ENE ≤/>2 mm) that should be examined and documented as it represents an additional prognostic factor. The methods applied (including ultrasound, CT, MRI) in the clinical diagnosing of ENE have limited sensitivity and specificity. In this regard initial attempts at computer-aided analysis of image data should be pursued. Furthermore, clinical diagnostic criteria should be standardized overall at the international level.

## Author Contributions

JK came up with the idea and planned the manuscript. AB and JK collected the data. AB drafted the manuscript and performed the systematic review and its analysis. AB provided the original data presented. SZ and KE provided expert opinion on histopathology and high resolution histopathologic pictures. IM helped with editing the manuscript. GP, CB, and JK reviewed the manuscript. All authors finally checked the manuscript and provided critical review of its content.

## Conflict of Interest

The authors declare that the research was conducted in the absence of any commercial or financial relationships that could be construed as a potential conflict of interest.
